# Regulation of Reactive Oxygen Species during Salt Stress in Plants and Their Crosstalk with Other Signaling Molecules—Current Perspectives and Future Directions

**DOI:** 10.3390/plants12040864

**Published:** 2023-02-14

**Authors:** Mahipal Singh Kesawat, Neela Satheesh, Bhagwat Singh Kherawat, Ajay Kumar, Hyun-Uk Kim, Sang-Min Chung, Manu Kumar

**Affiliations:** 1 Department of Genetics and Plant Breeding, Faculty of Agriculture, Sri Sri University, Cuttack 754006, India; 2 Department of Food Nutrition and Dietetics, Faculty of Agriculture, Sri Sri University, Cuttack 754006, India; 3 Krishi Vigyan Kendra, Bikaner II, Swami Keshwanand Rajasthan Agricultural University, Bikaner 334603, India; 4 Centre of Advanced Study in Botany, Banaras Hindu University, Varanasi-221005, India; 5 Department of Bioindustry and Bioresource Engineering, Plant Engineering Research Institute, Sejong University, Seoul 05006, Republic of Korea; 6 Department of Life Science, College of Life Science and Biotechnology, Dongguk University, Goyang 10326, Republic of Korea

**Keywords:** reactive oxygen species, superoxide dismutase, hydrogen sulfide, catalase, ascorbate peroxidase, nitric oxide, salt stress, omics, phytohormones

## Abstract

Salt stress is a severe type of environmental stress. It adversely affects agricultural production worldwide. The overproduction of reactive oxygen species (ROS) is the most frequent phenomenon during salt stress. ROS are extremely reactive and, in high amounts, noxious, leading to destructive processes and causing cellular damage. However, at lower concentrations, ROS function as secondary messengers, playing a critical role as signaling molecules, ensuring regulation of growth and adjustment to multifactorial stresses. Plants contain several enzymatic and non-enzymatic antioxidants that can detoxify ROS. The production of ROS and their scavenging are important aspects of the plant’s normal response to adverse conditions. Recently, this field has attracted immense attention from plant scientists; however, ROS-induced signaling pathways during salt stress remain largely unknown. In this review, we will discuss the critical role of different antioxidants in salt stress tolerance. We also summarize the recent advances on the detrimental effects of ROS, on the antioxidant machinery scavenging ROS under salt stress, and on the crosstalk between ROS and other various signaling molecules, including nitric oxide, hydrogen sulfide, calcium, and phytohormones. Moreover, the utilization of “-omic” approaches to improve the ROS-regulating antioxidant system during the adaptation process to salt stress is also described.

## 1. Introduction

During their life cycle, plants encounter diverse biotic and abiotic stresses, varying from salinity, drought, cold, flooding, heat, mineral deficiency or excess, and exposure to toxins [1]. Stress has a detrimental effect on growth and development, leading to reduced productivity in different staple food crops [2,3,4,5]. Salinity stress is the most severe abiotic stress and regulates complex phenotypic changes. It leads to an electrolyte imbalance by perturbing the metabolic activities of plants, thereby severely influencing their development and productivity worldwide [1,6,7]. A vast area of cultivated land is affected by this type of stress [8,9]. Salinity impacts plants via (a) the osmotic effect, (b) the ionic effect, (c) hormonal imbalance, (d) nutrient imbalance, and (e) the generation of ROS [9,10,11]. Plants are often exposed to multifactorial stresses, which may exhibit cumulative effects on plants owing to integrated signaling pathways [5,6,11].

**Figure 1:**
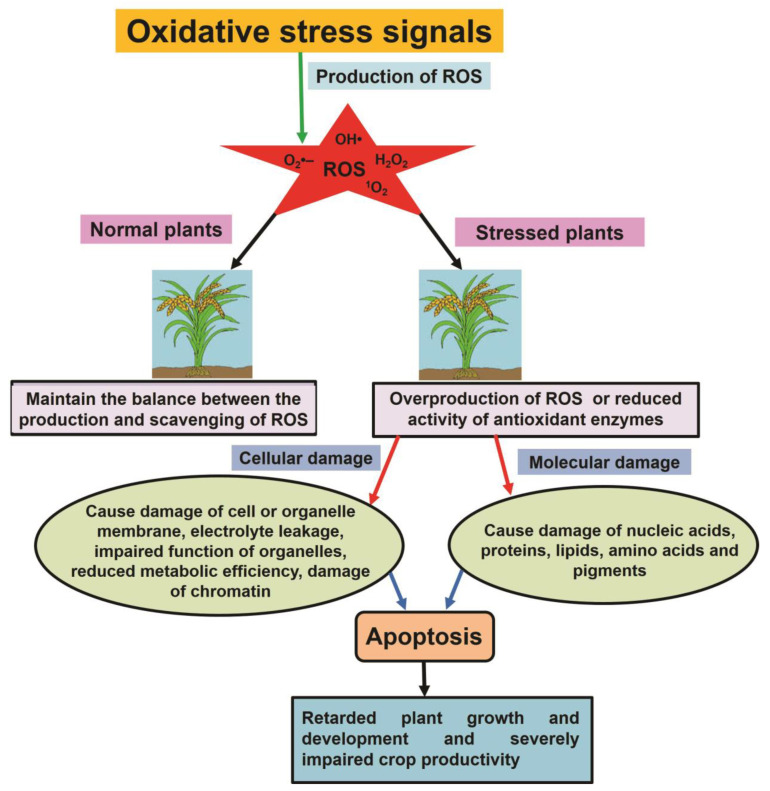
Excessive production of reactive oxygen species (ROS) in plants causes oxidative stress, leading to apoptosis, which severely affects plant growth and productivity. HO•, hydroxyl radical; H_2_O_2_, hydrogen peroxide; O_2_•−, superoxide radical; and ^1^O_2_, singlet oxygen.

ROS include singlet oxygen (^1^O_2_), superoxide radical (O_2_**^−^**•), hydroxyl radical (HO•), hydrogen peroxide (H_2_O_2_), alkoxyl radicals (RO•), and peroxy radicals (ROO•) (Table 1). They are generated under normal growth conditions [12,13]. However, extreme abiotic stress induces the overproduction of ROS [14], which alter cellular and molecular constituents, for instance, oxidizing DNA, proteins, carbohydrates, lipids, and enzymes and leading to programmed cell death (Figure 1) [15,16]. To prevent injuries, plants precisely control the generation of ROS through different enzymatic and non-enzymatic antioxidants. The enzymatic antioxidant plant defense machinery includes peroxidase (POD), superoxide dismutase (SOD), glutathione reductase (GR), catalase (CAT), dehydroascorbate reductase (DHAR), ascorbate peroxidase (APX), and monodehydroascorbate reductase (MDHAR), while non-enzymatic antioxidants include ascorbate (AsA), flavonoids, carotenoids, stilbenes, tocopherols, and other vitamins. These counteract oxidative stress either by restoring the level of endogenous antioxidants or by directly detoxifying the overproduced ROS, thereby increasing the tolerance to oxidative stress [17,18,19]. Unregulated production of ROS has a harmful effect on plant health. Conversely, physiological levels of ROS are involved in redox signaling, regulating plant development [20]. The precise regulation of ROS production facilitates communication between cells by amplifying the signals through nicotinamide adenine dinucleotide phosphate oxidase (NADPH) in response to various stresses by modulating the conformation of different proteins and triggering genes involved in stress tolerance [16]. Thus, elucidation and understanding of the molecular basis of ROS signaling and of the related downstream pathways would help alleviate stress in plants. Furthermore, considering the changing climate, the genetic manipulation of signaling pathways would further contribute to the development of stress-tolerant cultivars, minimizing stress-induced losses. In this review, we discuss the critical role of the antioxidant defense machinery that prevents the accumulation of ROS during salinity stress. Moreover, we also discuss the crosstalk with various signaling molecules and the use of genetic engineering to modulate salinity-related ROS signaling and to ensure optimal plant development and acclimation responses.

## 2. Production of ROS in Plant Cells

**Figure 2:**
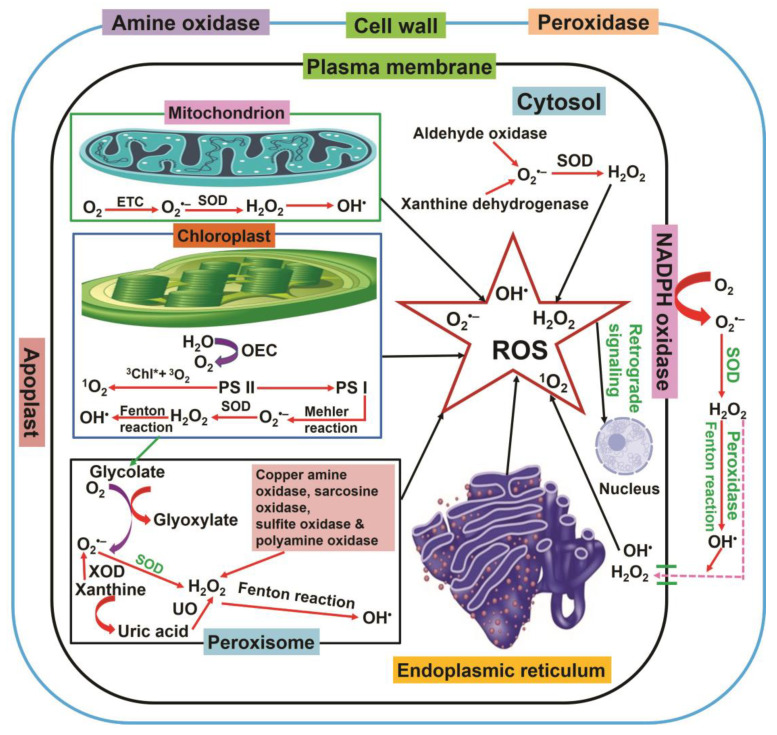
Mechanism and site of production of ROS in plants. SOD, superoxide dismutase; ROS, reactive oxygen species; ETC, electron transport chain; OEC, oxygen-evolving center; H_2_O_2_, hydrogen peroxide; PS I, photosystem I; PS II, photosystem II; O_2_^−^•, superoxide anion; ^1^O_2_, singlet oxygen; HO•, hydroxyl radical; NADPH, nicotinamide adenine dinucleotide; XOD, xanthine oxidase; and UO, urate oxidase.

The cell wall, plasma membrane (PM), peroxisomes, mitochondria, and chloroplasts are the primary sites of ROS generation in plant cells (Figure 2) [21,22]. Thus, compartmental production of ROS reflects its total production in plant cells [15,23]. Chloroplasts are the main site of ROS production, generating 30–100 times more ROS than mitochondria. Essentially, ROS are formed in photosystems I and II and in the electron transport chain (ETC). In the photosystems I and II, ROS formation is dependent on the reaction between light and chlorophyll (chl), an essential component of the photosystems [15,23,24]. Under high light, chl in photosystem II reaches a high-energy singlet excited state. The energy is further transferred to molecular oxygen, which is transformed into singlet oxygen. Some part of the energy is transmitted to the reaction center chlorophyll P680 and further to the ETC, a process known as photochemical quenching (pQ). Nevertheless, the absorbed energy might exceed the capacity of pQ; the surplus energy is dissipated as fluorescence or heat [25].

Carotenoids such as lutein and zeaxanthin extinguish triplet-excited Chl (^3^Chl*), inhibiting energy transmission to other molecules. If ^3^Chl* cannot be effectively quenched, it reacts with triplet oxygen, generating ^1^O_2_ [26]. Furthermore, after absorption of light energy, P680 reaches an excited state (^1^P680*), transfers an electron to pheophytin, and subsequently to a quinone in the PSII reaction center. In adverse conditions, if this quinone acceptor is not capable of taking any electrons, pheophytin couples with P680, forming the triplet photoexcited state ^3^P680* [27]. Theoretically, β-carotene in the PSII reaction center has the capacity to reduce ^3^P680*; however, the distance between β-carotene and ^3^P680* is too great, and the quenching is unsuccessful, leading to the production of ^1^O_2_ [28]. Additionally, some environmental stresses trigger the closure of stomata, reducing the level of carbon dioxide in the chloroplasts and leading to a severe reduction in the ETC. Thus, it increases the possibility of pairing between the quinone and ^1^P680* in PS II and enhances the production of ^1^O_2_ [29]. Conversely, PS I does not generate ^1^O_2_. However, photoreduction of the molecular oxygen at PSI results in the production of O_2_^−^• (Mehler reaction), which is further converted into H_2_O_2_ via superoxide dismutases (SODs) [30,31]. Then, metal ions such as Fe^2+^ transform H_2_O_2_ and O_2_^−^• into HO• [15]. In the roots, ROS are generated in the mitochondria, where electron leakage from complex I and III of ETC generates O_2_^−^•, which yields H_2_O_2_ via copper-zinc-SOD (CuZn-SOD) and manganese-SOD (Mn-SOD) [15,31]. In peroxisomes, the production of ROS is mainly catalyzed by glycolate oxidase [32]. Further, in the peroxisomal matrix, xanthine oxidase can catalyze the formation of uric acid and O_2_**^−^**•, the latter being converted to H_2_O_2_ through urate oxidase and SOD [33,34,35].

Apart from β-oxidation, H_2_O_2_ is produced in the peroxisomes by flavin oxidase and from disproportionation of O_2_**^−^**• [32,36]. Furthermore, copper amine oxidase, sarcosine oxidase, sulfite oxidase, and polyamine oxidase also produce H_2_O_2_ in the peroxisome [37]. MDHAR quenches H_2_O_2_ via the ascorbate-glutathione (AsA-GSH) cycle and produces AsA in the peroxisomes [38,39]. NADPH oxidase, quinine, lipoxygenases (LOX), POD, oxidases, germin-like oxalate, and reductase participate in ROS production in the apoplast [19,40]. Moreover, LOX, amine oxidases, and POD are the main sources of ROS in the cell wall [17,41], while quinone reductase and NADPH oxidase produce ROS in the PM [41,42]. Additionally, cytochrome P450 generates O^2−^• in the endoplasmic reticulum. β-oxidation, urate oxidase (UO), and glycolate oxidase generate H_2_O_2_ and O^2−^• in the glyoxysomes [43]. Finally, aldehyde oxidase (AO) and xanthine oxidase contribute to ROS production in the cytosol [44].

## 3. Role of Antioxidants in Oxidative Stress

**Figure 3:**
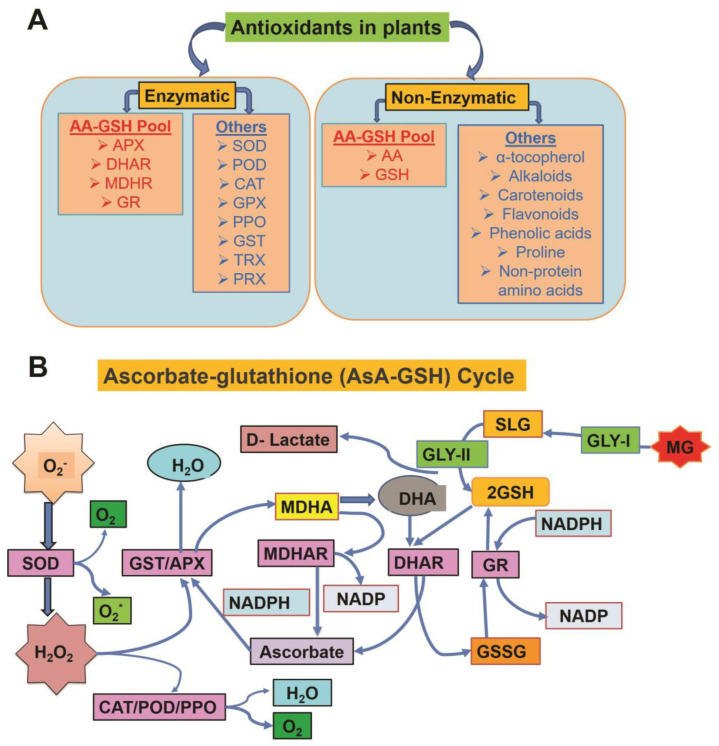
Antioxidant defense machinery in plants: (**A**) different types of antioxidants and (**B**) intricate regulatory mechanisms of enzymatic and non-enzymatic antioxidants. TRX, thioredoxin; SOD, superoxide dismutase; CAT, catalase; AA, ascorbic acid; AsA, ascorbate; APX, ascorbate peroxidase; DHAR, dehydroascorbate reductase; DHA, dehydroascorbate; GPX, glutathione peroxidase; H_2_O_2_, hydrogen peroxide; GSH, reduced glutathione; GR, glutathione reductase; GSSG, oxidized glutathione; MDHAR, monodehydroascorbate reductase; MDHA, monodehydroascorbate; GST, glutathione S-transferase; NADPH, nicotinamide adenine dinucleotide phosphate; DHA, dehydroascorbate; AsA, ascorbate; AA, ascorbic acid; AsA-GSH, ascorbate-glutathione; GLA-I, glyoxalase-I; GLA-II, glyoxalase-II; PPO, polyphenol oxidase; POD, peroxidases; PRX, peroxiredoxins; O_2_^−^•, superoxide anion; ^1^O_2_, singlet oxygen; HO•, hydroxyl radical.

In plant cells, physiological ROS function as secondary messengers, being redox-signaling molecules in various signaling pathways that regulate the response to adverse environmental conditions [41,45]. In conditions of extreme stress, excess production of ROS is toxic and leads to programmed cell death [14]. The type of action of ROS (signaling, regulatory, or destructive) is determined by the balance between their generation and quenching [46]. The enzymatic and non-enzymatic antioxidants counteract excess ROS (Figure 3A,B), maintaining adequate concentrations in different compartments of plant cells [47,48]. Increasing the level of antioxidant enzymes via exogenous supply or through genetic engineering could reinforce the plant defense system, enabling plants to adapt under various stress conditions.

### 3.1. Enzymatic Antioxidants

The antioxidant enzymes—including CAT, SOD, MDHAR, APX, guaiacol peroxidase (GPX), DHAR, and GR—localized in diverse plant cell compartments comprise the plant antioxidant defense system (Figure 3A). In the following sections, we will discuss each antioxidant enzyme in detail.

#### 3.1.1. SOD

SOD (E.C.1.15.1.1) is a metalloenzyme present in all aerobic organisms. It represents the first bulwark against ROS-induced damage owing to different environmental stresses. SOD catalyzes the conversion of O_2_^−^• into O_2_ and H_2_O_2_, ensuring its elimination [49]. Thus, this enzyme decreases the formation of HO• via the Haber–Weiss reaction [50]. Based on the type of prosthetic metal, SODs in plants are classified into three isoenzymes: iron-SOD (Fe-SOD), CuZn-SOD, and Mn-SOD. These are common in various organelles, including mitochondria, chloroplasts, the nucleus, apoplasts, and peroxisomes [51]. SOD isoenzymes catalyze the dissociation of O^2−^• into H_2_O_2_ and O_2_ as follows:

O_2_^•−^+ SOD − M^2+^ → O_2_ + M^+^--------Step 1
O_2_^•−^+ 2H^+^-SOD − M → H_2_O_2_ + M^2+^-------Step 2

The overall reaction is presented as the following equation:

O_2_^•−^+ O_2_^•−^+ 2H^+^ → 2H_2_O_2_ + O_2_

Superoxide free radicals are produced at any location of the ETC; hence, SOD activation is evident in mitochondria, peroxisomes, and chloroplasts [52]. The Haber–Weiss reaction is ten thousand times faster than the natural process [14].

The abundance of different SOD isoenzymes in plants is influenced by climatic conditions and the growth phase of the plant [50]. Compounds such as ABA influence the expression of antioxidant enzymes such as GR, APX, and SOD [53]. Corpas et al. [54] noted that SOD has inconsistent transcript-level expression, which varied between cell types. SOD compartmentalization and activation in different cell structures are essential for the protection of plants against abiotic stress-induced oxidative stress as well as for the optimal development of plant signaling [55]. H_2_O_2_ and molecular oxygen are both byproducts of the enzymatic reaction catalyzed by SOD. H_2_O_2_ regulates different signaling activities. It is an essential signal during plant-microbe interactions, the response to inflammation, the closing of the stomata, and osmotic and excessive light stress [56]. Abiotic stress leads to SOD upregulation in different plant species [57].

#### 3.1.2. CAT

CAT (E.C.1.11.1.6) is a tetrameric iron-containing redox enzyme present in all organisms exposed to O_2_ [58]. This enzyme catalyzes the conversion of H_2_O_2_ into water and O_2_. The iron prosthetic group of the enzyme is essential for its catalytic activity. This enzyme shows higher reduction activity toward H_2_O_2_ than toward organic peroxides (R-O-O-R). CAT eliminates H_2_O_2_ with minimal energy use [59] and a very high rate of conversion (6 × 106 molecules per min). It has a reaction pattern highly distinct from other antioxidant enzymes as it does not need a reducing agent [58]. β-oxidation, photorespiration, oxidative stress, and purine catabolism are the processes that produce most H_2_O_2_ [51]. Based on subunit size, function, structure, type of prosthetic groups, and sequences CAT is classified into three main groups: monofunctional CAT (group-1), catalase-peroxidase (group-II), and nonheme CAT (group-III) [60]. Most of the plants possess group I CAT, which is widely dispersed in different compartments of the plant cell, such as the cytosol, chloroplasts, and mitochondria [61]. However, angiosperms contain all three different isoforms of CAT.

CAT1 is critical for the elimination of H_2_O_2_ produced during photorespiration and is widely present in pollen and seeds [62], but less so in roots. CAT2 is prevalent in typical photosynthetic cells. This isoform protects different cells from oxidative stress damage. CAT3 is widely found in vascular tissues and leaves and scavenges H_2_O_2_ produced by the β-oxidation of lipids in the glyoxysomes [63]. Stressful conditions require higher energy generation and expenditure. Subsequently, catabolism increases, leading to the generation of higher amounts of H_2_O_2_ [64].

CAT isoforms act in two modes: catalytic and peroxidatic. In the former, they catalyze the direct conversion of H_2_O_2_ to water and O_2_ or other oxidized compounds such as lower alcohols (methanol, ethanol), aldehydes (formaldehyde), etc. In the latter, they convert lower amounts of H_2_O_2_ by oxidizing various hydrogen donors such as small-molecule alcohols, aldehydes, and ascorbic acid (AA) as follows [65]:

RH_2_ + H_2_O_2_ → R + 2H_2_O
CAT-Fe-OOH + C_2_H_5_OH → CAT-Fe-OH + H_2_O + CH_3_CHO (peroxidatic reaction)
CAT-Fe-OOH + H_2_O_2_ → CAT-Fe-OH + H_2_O + O_2_ (catalytic reaction)

Thus, CATs generate useful compounds and water from harmful compounds and H_2_O_2_. Depending on the reaction mode, catalases may be further classified as

HPI catalase: comprises isoenzymes with dual function, participating in both catalytic and peroxidative reactions.

HPII catalase: comprises isoenzymes that only participate in peroxidative reactions; these CATs play a crucial role in protecting plants from abiotic stress caused by different factors [66]. Plants exposed to normal levels of abiotic stress factors such as ozone, sulfur dioxide, and UV-B radiation presented a quick reduction in the expression of CAT1 and a rapid increase in the expression of CAT2.

Gondim et al. [67] reported that in corn, H_2_O_2_ pretreatment enhanced CAT activity and prevented the deleterious effects of salinity. Furthermore, a higher CAT activity was essential for the existence of plants under reasonable metal stress. However, Spartan metal stress causes the permanent impairment of CAT. The function of CAT is taken over by other enzymes, which ensure abiotic stress is managed [68].

#### 3.1.3. APX

APX (E.C.1.1.11.1) is a key enzyme found abundantly in plants and algae [69]. It is distributed in five different cellular components: stomata (sAPX), thylakoid membrane (tAPX), the membrane (mAPx) and cytosol (cAPX) of microbodies, and mitochondria (mitAPX) [70]. APX is present in every part of the plant and scavenges H_2_O_2_ through the AsA-GSH pathway (or Asada–Halliwell–Foyer pathway) [71]. APX mostly scavenges H_2_O_2_ in the cytosol and chloroplasts, while CAT primarily acts in the peroxisomes. APX, in the presence of a reducing agent such as AA, reduces H_2_O_2_ to water while the other reactant is oxidized (e.g., AA to dehydroascorbate, DHA). This enzyme is very unstable in the absence of AA. At concentrations AA < 20 µM, the activity of APX drastically decreases [72]. Under stress conditions, APX is a more effective H_2_O_2_ scavenger than CAT because it is more extensively distributed and has a stronger affinity for H_2_O_2_. APX acts as an antioxidant by the following reaction:

H_2_O_2_ + AA→2H_2_O + DHA

*To provide significant protection during oxidative stress, the enzymatic and non-enzymatic antioxidant molecules closely coordinate their actions. By keeping the redox equilibrium under stress, APX connects the two pathways. Furthermore, it has a particular affinity for H_2_O_2_ [71]. Plants lacking APX1 have delayed growth and development. In the absence of APX1, H_2_O_2_ levels increase and cause aberrant stomatal closure [73]. In APX1-deficient plants, light stress results in H_2_O_2_-mediated-enhanced activation of heat shock proteins [51,74]. Furthermore, APX and CAT at least partially complement each other’s shortcomings [75]. Moreover, the activities of SOD, CAT, reduced glutathione (GSH) reductase, and APX typically increase in response to various environmental stresses [72].*

#### 3.1.4. MDHAR

MDHAR (E.C.1.6.5.4) restores AA from MDHA in the presence of NADPH as a reducing agent [76]. Subsequently, AA concentrations in plant cells return to normal. MDHAR co-exists with APX in different organelles, as APX scavenges H_2_O_2_ and concomitantly oxidizes AA [51]. MDHAR has several isoforms that are present in organelles such as mitochondria, chloroplasts, glyoxysomes, peroxisomes, and the cytosol [77].

MDHA + NADPH→AA + NADP^+^

The monovalent oxidation of AA results in MDHA. In the absence of MDHAR, MDHA will convert non-enzymatically to AA and DHA. Then, DHA is converted to AA by DHAR as GSH oxidizes [78]. A rapid regeneration of AA is essential for its function as an antioxidant. As the restoration of AA levels is controlled by NADPH-dependent MDHAR, this enzyme is critical for conserving an optimal level of AA and thus its antioxidant function [79].

#### 3.1.5. DHAR

DHAR (M.C.1.8.5.1) is an enzyme that catalyzes the reduction of DHA to AA in the presence of GSH as an electron contributor [78]. Together with MDHAR, DHAR ensures the renewal of the cellular AA pool. DHAR regulates AA levels in the symplast and apoplast of plant cells and simultaneously maintains the redox capacity of the cells [80]. It is found in high amounts in green, etiolated shoots, roots, and seeds [81]. Isoforms DHAR1 (in the cytosol) and DHAR3 (in the chloroplasts) play similar roles and account for nearly all the enzymatic activity. Conversely, DHAR2 is only a minor contributor to the overall antioxidant action [82].

DHA + 2GSH→AA + GSSG

The catalytic mechanism of DHAR involves three steps [83]. In the first step, DHA is attached to the catalytic cysteine molecule of DHAR by nucleophilic substitution (DHAR-S–). In the second step, the reactive cysteine molecules in sulfonic form bind to GSH and produce various disulfides (DHAR-S-SG). Consequently, GSH is deprotonated to GS–. In the final step, the GS– residue attached to the active cysteine is eliminated via nucleophilic substitution. Thus, a catalytic cysteine molecule is reduced, and one glutathione disulfide (GSSG) molecule is released. Together with the other antioxidant enzymes, such as SOD, APX, MDAR, and GR, DHAR ensures the completion of the Foyer-Asada-Halliwell pathway and the removal of ROS in chloroplasts and cytoplasm [84]. Furthermore, DHAR overexpression improves the tolerance of plants to various types of abiotic stress, such as exposure to ozone, high salinity, and water scarcity [85,86].

#### 3.1.6. GR

GR (E.C.1.6.4.2) belongs to the oxidoreductase class of enzymes and needs NADPH as a reducing agent to convert GSSG to GSH. The isoforms of GR are abundant in chloroplasts, cytosol, and mitochondria [87]. In various plant species, GR exists mostly as a homodimer; however, it may also exist as a monomer or heterodimer. Its conversion to a tetrameric form or other higher molecular mass aggregates depends more on pH and temperature than on the amount of reactants or product [88]. GSH is effectively involved in the restoration of AA levels from MDHA and DHA, which subsequently leads to the transformation of GSH to its oxidized state, GSSG. The same amount of NADPH is required for both the reduction of GSSG to GSH and the formation of GSSG [89]. GR is a vital enzyme for the AsA-GSH cycle and involves the establishment of a bond between two sulfur atoms in two molecules of glutathione [90]. The three isoforms of GR are situated in the mitochondria, chloroplasts, and cytosol of plant cells [91]. The exposure of plants to diverse agroclimatic conditions determines the development of different isoforms of GR [92]. The activity of GR depends on the availability of the substrate, and this factor influences extremely the redox changes in the GR. The reduced state of GR demonstrates higher resistance than the oxidized conditions [93]. GR is essential for cell protection against ROS by converting GSSG to GSH with the complementary oxidation of NADPH and restoring GSH concentration [94]. It is mainly found in chloroplasts; however, small quantities are also present in the cytosol and mitochondria. GSH plays an essential role in preventing protein thiol groups from getting oxidized and also counteracts HO• and ^1^O_2_ [8].

GSSG + NADPH → 2GSH + NADP^+^

GR exists mostly as a homodimer with a molecular weight ranging from 100 to 150 kDa. It comprises a single FAD molecule per monomer. In the absence of thiols, GR tends to aggregate into tetramers and even larger forms [95]. The catalytic activity of GR consists of two stages: first, the flavin molecule is reduced by NADPH. Simultaneously, the flavin is oxidized, and a redox-active disulfide bond is reduced to form a thiolate negative ion and a Cys molecule. Second, GSSG is reduced through a thiol disulfide interchange. Similarly, GR controls GSH/GSSG quantities and facilitates the formation of GSH for DHAR and GPX. The DHAR and GPX convert H_2_O_2_ and DHA to H_2_O and AA [96]. An enhanced activity of GR denotes stress leniency and might change the redox nature of significant constituents of the ETC. Such enhanced activity was reported under temperature, metal, salt, and drought stress in different plant varieties [16]. The GR enzyme is also responsible for plant adaptation and signaling during cold stress. Likewise, plant resistance to lower temperatures was associated with greater GR activity in cereal crops [97].

#### 3.1.7. GPX

GPX (E.C.1.11.1.7) is composed of 40–50 kDa molecular weight monomers. It actively decomposes H_2_O_2_ generated by regular metabolism and by exposure to different stress conditions. Hence, it is also called the “stress enzyme” [98]. It contains Ca^2+^ and four disulfide bridges. It is dynamically involved in the biological production of lignin and contributes to the defense against biotic stress by breaking the indole acetic acid (IAA) and consuming the H_2_O_2_ in the process [99,100]. Plants contain GPX in various tissues and subcellular compartments and during the numerous stages of development. GPX is mostly localized in the chloroplast; however, various isoforms are located in the mitochondria, cytosol, and peroxisomes [101]. This enzyme contains cysteine in the active site and actively transforms H_2_O_2_ and lipid peroxides to water and fatty alcohols, using GSH as the cofactor. Subsequently, GSH is oxidized to GSSG. The enzyme GPX possesses cysteine in the active location instead of selenocysteine; thus, GPX may present selenium-dependent and -independent forms [102].

The GPX enzyme is considered the primary enzyme in the elimination of H_2_O_2_ as it functions both outside and inside the cell, in the organelles (the cytosol and vacuole).

H_2_O_2_ + GSH → H_2_O + GSSG

GPX is engaged in different biosynthetic pathways, such as the synthesis of lignin, which contributes to cell wall strengthening, the catalytic breakdown of IAA, and the production of ethylene (ET). It contributes to the rehabilitation of the biotic and abiotic stress-induced lesions. In plant cells, its activity is enhanced by exposure of the plant to different stress factors [103]. Around 90% of the peroxidase activity in plants is attributed to GPX. Conversely, Mika and Luthje [98] showed that exposure of plants to other types of environmental stress, such as heavy metals, high salinity, and ozone, does not increase the activity of GPX.

### 3.2. Non-Enzymatic Antioxidants

The non-enzymatic antioxidants, which include AA, GSH, alpha-tocopherol, carotenoids, different phenolics, flavonoids, and the amino acids and osmolyte proline, make up the other half of the antioxidant machinery in plants. They play a crucial role in plant development and growth by adjusting cellular processes including cell division, cell expansion, maturity, and death. Moreover, they serve to protect various cell components from injury [104].

#### 3.2.1. AA

AA is the most prevalent, water-soluble, and extensively researched antioxidant agent [105]. Approximately 40% of the AA is found in the stomatal regions and chloroplasts, with concentrations reaching 20–300 mM. Approximately 90% of the AA is localized in the cytosol, and only small amounts are localized in the apoplast. Thus, AA is the first line of defense against ROS damage [106]. In plants, AA is mainly produced in the mitochondria via the Smirnoff–Wheeler pathway, catalyzed by L-galactano-γ-lactone dehydrogenase. The remaining amount is formed from D-galacturonic acid [107]. Ascorbate is transported from the mitochondria to other cell organelles by facilitated and passive diffusion [108]. AA regulates various physiological functions, including plant growth, diversification, and metabolism [109].

The highest concentration of AA is found in the leaves. Furthermore, a linear relationship exists between the amounts of chlorophyll and AA. AA represents one of the most effective antioxidant agents; it counteracts ROS by donating electrons in different enzymatic and non-enzymatic pathways. AA directly scavenges H_2_O_2_, HO•, and O_2_**^−^**• and restores α-tocopherol from tocopheroxyl free radicals produced through metabolism or induced by various types of stress. Thus, it contributes to the protection of cell membranes and the regeneration of the lipoproteins in their structure [110]. AA also plays a crucial role in the AsA-GSH cycle, maintaining the metal ions of the related enzymes in a reduced form to exert their activity [111]. The first stage in the two-step oxidation of AA is the oxidation of MDHA, which, if not immediately converted to ascorbate, non-enzymatically transforms to AA and DHA. AA in its reduced state functions as the cofactor of violaxanthine de-epoxidase, ensuring the disposal of the surplus of excitation energy [112]. Additionally, AA has been linked to the synthesis of zeaxanthine and the pH-mediated control of PSII activity, preventing photo-oxidation [113]. Amplification of *MDAR* increased the tolerance of tobacco plants to ozone, high salinity, and polyethylene glycol. This was attributable to the subsequent increase in AA levels [78]. Furthermore, drought and an increased intensity of light dramatically boosted the amount of AA in plants [114].

#### 3.2.2. GSH

Nearly all subcellular compartments, including the mitochondria, chloroplasts, vacuoles, endoplasmic reticulum, peroxisomes, cytosol, and apoplasts, contain high amounts of glutathione, a hydrosoluble antioxidant thiol tripeptide (γ -glutamyl-cysteinyl-glycine) [115]. Among the different cell organelles, the highest concentrations of glutathione are observed in chloroplasts (1–4 mM) [116]. In its reduced state, glutathione comprises three amino acid residues: cysteine, glutamic acid, and glycine [117]. Owing to its high reduction potential, GSH is involved in various reactions [118] and participates in diverse biological processes, such as cell proliferation, differentiation, senescence, and death; control of sulfate transport; xenobiotic detoxification; conjugation of metabolites; regulation of the enzymatic activity; synthesis of nucleotides, amino acids, and phytochelatins; and regulation of the expression of stress-responsive genes [119]. GSH is the main source of non-protein thiols for most plant cells; its thiol group makes GSH especially suited for a broad number of metabolic tasks. The nucleophilic character of the thiol group is crucial for the interaction with minerals and generation of mercaptide, as well as for the reaction with specific electrophiles [120].

GSH is one of the most important antioxidants, as it helps maintain a low level of ROS [111]. It acts as an antioxidant through various mechanisms. Thus, GSH is essential in the antioxidant defense as it can replenish AA through the AsA-GSH cycle [121]. GSH directly scavenges different ROS, such as ^1^O_2_, O_2_**^−^**•, HO•, and H_2_O_2_, protecting diverse biomolecules. Subsequently, glutathione-derived derivatives, or GSSG, are formed as byproducts. GSH also plays an important role in reproducing AA to yield GSSG using NADPH as the reducing power [111]. The cellular supplies of GSH are restored either by the conversion of GSSG by GR or through de novo synthesis. Besides acting as an enzymatic co-substrate and reducing aging, GSH might sense redox changes and convey them to specific target proteins [122]. ROS-induced modifications of proteins may impact metabolism directly (by specific redox modifications of key amino acids) or indirectly (by early redox-reliant alterations of TFs and subsequent changes in gene expression). Besides chelating heavy metal ions and counteracting the formation of ROS in plants, GSH also participates in the production of phytochelatins by phytochelatin synthase [123]. Thus, the fine equilibrium between the levels of GSSG and GSH is essential for preserving the redox homeostasis of the cell.

#### 3.2.3. α-Tocopherol

Among the four existing isomers (α-, β-, γ-, δ-), α-tocopherol has the strongest antioxidant properties. Tocopherols are exclusively produced by photosynthetic organisms and are only found in the fresh tissues of plants [124]. α-tocopherol is converted to γ-tocopherol by γ-tocopherol-methyl-transferase, an enzyme encoded by *VTE4* [116]. Tocopherols are a group of lipophilic antioxidants that effectively remove ROS and fatty radicals; thus, they are vital elements and important defenders of cell membranes [125,126]. The major function of α-tocopherol is to prevent photo-oxidative damage. It decreases the levels of singlet oxygen through a charge transfer mechanism [120]. By interacting with oxygen and quenching its surplus energy, tocopherols are renowned for their capacity to protect fatty molecules and related organelle barriers, as well as the structure and function of the PSII center [127]. Additionally, tocopherol effectively traps free radicals, preventing the initiation of lipid peroxidation [128]. At the membrane-water interface, it reduces the lipid radicals RO*, ROO•, and RO•, transforming into a free radical (TOH•) in the process. TOH• interacts with GSH and AA, which convert into its reduced form [129]. Yu and Tang [130] found that one molecule of α-tocopherol can successfully counteract 120 singlet oxygen molecules. Additionally, α-tocopherol acts as a chain reaction inhibitor, scavenging the free radicals produced during the oxidation of polyunsaturated fatty acids [131]. Subsequently, α-tocopherol is converted to a tocopheroxyl radical that is converted back into α-tocopherol through interactions with several other antioxidants [129]. Tocopherols play a large role in several different systems that prevent the oxidation of polyunsaturated fatty acids. Various abiotic stressors increase the amount of α-tocopherol in the tissues of photosynthetic plants [128].

#### 3.2.4. Carotenoids

Carotenoids, a class of phytonutrients generated by plants, algae, certain bacteria, and fungi, are pigments also known as tetraterpenoids [132]. They are located in the chloroplasts and are a class of lipophilic antioxidants [133]. There are more than eleven thousand carotenoids. They have been mainly divided into two groups: xanthophylls (oxygen-containing carotenoids) and carotenes (hydrocarbon carotenoids). All carotenoids are tetraterpene derivatives, being formed from eight isoprene units and having a total of forty carbon atoms [134]. They are members of a class of molecules called sensors that collect light between 450 and 570 nanometers and send that energy to the chlorophyll molecule [135]. Carotenoids have a long conjugated alkyl chain, which makes them fat-soluble. They are the most prevalent lipid-soluble antioxidants and play a critical role in protecting lipoproteins and cell membranes from lipid peroxidation. They scavenge peroxyl radicals more effectively than any other ROS [136]. The peroxyl radicals are reduced by carotenoids, leading to the formation of a resonance-stabilized carbon-centered radical intermediate. Among the carotenoids, lycopene, and carotene are the most prevalent and effective antioxidants [137]. Owing to the substantial number of conjugated double-bonds in trans-configuration that its molecule contains, carotene is a notably potent detoxifier of the singlet oxygen molecule. The expanded conjugated framework in carotenoids is highly reducing, allowing easier extraction of hydrogen atoms from the allylic sites and facilitating free-radical addition processes [138]. Lycopene lowers the level of peroxyl radicals through electron transfer, creating an inert, resonance-stabilized carbon-centered radical [139]. β-carotene has an excellent ability to neutralize ^1^O_2_ without deterioration. The chemical reactivity of β-carotene with free radicals, such as O_2_**^−^**•, HO•, and ROO•, arises from the conjugated double-bonded structure, which allows the delocalization of unpaired electrons and is the primary cause of its antioxidant capacity [134]. Higher concentrations of carotenoids protect lipids from peroxidative injury [140]. Carotenoids demonstrate their antioxidative action in the photosynthetic equipment in four ways: (a) they react with lipid peroxidation products to end chain reactions; (b) they remove ^1^O_2_, which produces heat as a by-product; (c) they inhibit the formation of ^1^O_2_ by counteracting 3Chl* and Chl*; and (d) they release energy when it exceeds the required amount through the xanthophyll cycle [141].

#### 3.2.5. Flavonoids

Flavonoids are mostly distributed throughout the plant world and are particularly abundant in the leaves, floral organs, and pollen [142]. Based on their structural characteristics, flavonoids can be divided into four groups: flavones, flavonols, anthocyanins, and isoflavones. They are largely responsible for the coloring of flowers, fruits, and seeds and are important for plant fertilization, pollen propagation, and defense against phytopathogens [143]. Over 8000 flavonoid derivatives were identified and analyzed. The main structural component of flavones is a backbone consisting of two phenyl rings connected by a heterocyclic ring [144]. Compared with flavones, flavonols contain an additional hydroxyl group [145]. Quercetin is the most prevalent polyphenolic flavonoid. It protects DNA from the oxidative degradation caused by H_2_O_2_, HO•, and O_2_^−^•. Furthermore, anthocyanidin acts as a strong inhibitor of fatty acid oxidation [146]. The ability of anthocyanidin to chelate metal ions has been linked to its ability to reduce fatty acid peroxidation [147]. Due to their ability to prevent highly energetic wavelengths from reaching ROS-generating cells, flavonoids were thought to be a supplemental ROS-neutralizing system in plants suffering from injuries to the photosynthetic apparatus. Furthermore, flavonoids scavenge ^1^O_2_, reducing its damaging consequences on the external layers of the chloroplasts. The concentration of flavonoids in plant cells typically exceeds 1 mM. Various flavonoids act as possible regulators of lipoxygenase, the enzyme that converts polyunsaturated fatty acids into corresponding peroxides and hydroperoxides [148]. Owing to their advantageous reduction potential, flavonoids are extremely effective neutralizing agents for H_2_O_2_ and good inhibitors of lipid oxidation. This is one of the most actively researched features of flavonoids.

#### 3.2.6. Proline

Proline is a potential non-enzymatic antioxidant that counteracts the negative consequences of various ROS in the components of different plant species [149]. Proline is synthesized from glutamic acid via the intermediate pyrroline 5-carboxylate. In plants, two enzymes, pyrroline-5-carboxylate reductase and 1-pyrroline-5-carboxylate synthetase, catalyze this process [150]. Proline can prevent lipoperoxidation-related damage and is an effective scavenger of HO• and ^1^O_2_. In plants, it builds up significantly under stress, either as a result of enhanced production or decreased breakdown [151]. Plants respond to external and internal stimuli by accumulating free proline. Szabados and Savouré [152] reported the diverse roles of proline in plant cells. Owing to its properties as a scavenger of singlet oxygen molecules and hydroxyl radicals, proline is essential for avoiding ROS-induced oxidative impairment. Proline may relieve stress on DNA, various cell barriers, and protein complexes throughout the recovery process. Additionally, following stress reduction, it serves as a source of nitrogen and carbon for growth [41]. Proline biosynthesis contributes to the modulation of cellular redox potential as well as the storage and transportation of energy. An increase in proline synthesis is highly beneficial for plants’ lenience to ecological stresses. However, the literature on the relationship between the metabolism of ROS and proline is scarce.

## 4. Downstream Redox Signaling during Oxidative Stress in Plants

Redox reactions are ubiquitous in all living organisms and regulate the generation of ROS in plant cells [153]. The existence of an equilibrium between the generation of ROS and their eradication by the antioxidant enzymes is critical for maintaining an appropriate level of ROS in plant cells [154]. This level of ROS is below the cytotoxic concentration and is essential for precise redox signaling [9,19]. Plant scientists use the term “redox biology” for ROS acting as a signaling molecule to regulate and maintain fundamental cellular processes in plants [19,21,153,154]. Redox signals maintain the homeostasis between basal levels of ROS, which function as signals to stimulate downstream signaling pathways that regulate diverse cellular processes; however, increased concentrations of ROS cause oxidative damage [153]. Hence, a steady equilibrium between ROS production and their quenching dynamically coordinates redox sensitive constituents activating specific downstream cascades [155]. Conversely, environmental stresses may disrupt the balance between the production and scavenging of ROS by antioxidants, leading to the overaccumulation of ROS and oxidative stress [16]. Oxidative stress damages nucleic acids, proteins, and lipids and interferes with carbohydrate metabolism, leading to cell dysfunction and death.

## 5. Oxidative Stress under Salt Stress

Exposure of plants to several abiotic stresses under field conditions resulted in the generation of oxidative stress via the overproduction of ROS. ROS are produced mainly in chloroplasts and, to a lesser extent, in PMs, mitochondria, apoplasts, and peroxisomes [15,23]. Most of the environmental stresses affect the availability of CO_2_ and inhibit its fixation, resulting in a decrease in molecular oxygen and an overgeneration of ROS. The activity of the chloroplasts is impaired, thereby hindering the process of photosynthesis [14]. However, the production of ROS significantly differs between different plant species, cultivars, stress types, stress tolerance levels, and the time of exposure to stress. High salinity stress affects plants by altering numerous cellular processes and inducing osmotic stress, ion toxicity, genotoxicity, and nutritional deficiency, leading to overgeneration of ROS [6]. Rehman and colleagues [156] reported that, compared with control plants, plants exposed to high salinity stress (NaCl 100 and 200 mM) presented higher levels of H_2_O_2_ and a 2- to 3-fold increase in the levels of thiobarbituric acid. Furthermore, the impact of the salinity stress on the generation of ROS differed between different developmental stages and tissues. For example, the root is more sensitive to salinity-induced oxidative stress than young and mature leaves. Cheng et al. [157] found that in rice, compared with a control, salinity stress increases the total electrolyte leakage, lipid peroxidation, and ROS levels more than 2-fold in the root. Additionally, Ahanger et al. [158] showed that in tomato, compared with a control, salinity stress increases oxidative stress by increasing the levels of H_2_O_2_ and O_2_^−^• (176% and 157%, respectively), electrolyte leakage (158%), and malondialdehyde (MDA, 94%). Compared with control, salinity stress induced a 2-fold increase in electrolyte leakage and MDA levels in sweet peppers and in the levels of electrolyte leakage, MDA, H_2_O_2_, and O_2_^−^• in mung bean (100 mM NaCl) [159]. Furthermore, compared with control, maize exposed to 120 mM NaCl also presented increased levels of MDA (25%) and H_2_O_2_ (50%) [160]. The degree of oxidative stress differed between different genotypes of the same plant species. When two sunflower genotypes, FH-572 and FH-621, were exposed to 120 mM NaCl, the level of H_2_O_2_ increased by 78% in FH-572, whereas it reduced by 20% in FH-621, indicating that FH-621—not FH-572—is a salt-tolerant genotype [161]. Similarly, when two sesame genotypes, TS-5 and TH-6, were exposed to 70 mM NaCl, TS-5 showed better salt tolerance than TS-6 [162]. Moreover, Mhadhbi et al. [163] revealed a genotype-dependent association between salt tolerance and ROS content (H_2_O_2_ and MDA) during high salinity stress in *Medicago truncatula*. *Ailanthus altissima* exposed to 150 mM NaCl had a higher level of antioxidant activity than non-stressed *Ailanthus altissima*. However, the level of H_2_O_2_ did not differ significantly between the two groups, indicating a connection between the antioxidant defense machinery and the enhanced insensitivity of plants in harsh environmental conditions [164]. Collectively, these studies demonstrated that plants might respond differently to salt-induced oxidative stress.

## 6. Antioxidant Defense System in Plants under High Salinity Stress

In adverse environmental conditions, plants activate their antioxidant defense machinery to minimize the damage induced by oxidative stress. The antioxidant defense response differs depending on the species, genotype, stress type, and duration of exposure to stress. Various plant species have different methods to increase their antioxidant capacity. Regulation of the antioxidant defense system counteracts the effects of high salinity stress and increases stress tolerance in various plant species. The response and the potency of the antioxidant activity vary depending on the developmental stage, type of tissue, salinity degree, and duration of exposure to stress [165].

For example, Vighi et al. [166] reported that the salt-tolerant (BRS Bojuru) and the salt-sensitive (BRS Pampa) rice cultivars have distinct responses to high salinity stress.

OsSOD3-Cu/Zn, OsGR2, OsGR3, and OsAPX3 are the distinct markers of the salt-tolerant rice cultivar, not the salt-sensitive one. Furthermore, the salt-tolerant wheat cultivar Suntop presented higher activities of the antioxidant enzymes CAT, APX, SOD, POD, and GR than the salt-tolerant wheat cultivar Sunmate. Additionally, the barley salt-tolerant cultivar, CM72, presented higher activities of the antioxidants than the sensitive cultivar. These results indicate that an elevated level of antioxidants or enhanced antioxidant activity contributes to salt tolerance in plants [167]. Another study reported that in salt-stressed *Vicia faba*, when the levels of H_2_O_2_ exceeded 90%, the expression of CAT, SOD, and GR at the mRNA level and that of AsA at the protein level were elevated, demonstrating the regulation of the antioxidant response by high salinity and its mitigation [168].

The role of the antioxidant “defense system” in improving salinity tolerance was demonstrated by using natural or chemical protective compounds to regulate the antioxidant activity. For instance, Alsahli et al. [169] reported that salicylic acid (SA) application resulted in a 2-fold increase in APX, CAT, and SOD activity and a 3-fold reduction in H_2_O_2_ level in salt-stressed wheat seedlings (compared with not-stressed wheat seedlings). Similarly, combinatorial treatment with humic acid and jasmonic acid (JA) also enhanced APX activity in sorghum, improving its salt tolerance [170]. In salinity-stressed sour orange, supplementation with polyamines enhanced the antioxidant response [171]. Furthermore, in salinity-stressed wheat (100 mM NaCl), nitrogen enhanced SOD, APX, GR, CAT, MDHAR, and DHAR activities and GSH and AsA biosynthesis, decreased H_2_O_2_ levels (2.5-fold), and increased O_2_^−^• levels (1.7-fold) [172]. Exogenous application of silicon also increased the antioxidant response while reducing electrolyte leakage and MDA and ROS levels in salinity-stressed mung beans [159]. In soybean, silicon upregulated GmAPX1 (8-fold), GmCAT1 (3-fold), and GmCAT2 (4-fold), resulting in enhanced tolerance to salinity stress [173]. Similarly, in salinity-stressed strawberries cultivated in hydroponic conditions, the transcript levels of CAT, cAPX, Mn-SOD, and GR decreased 0.4-fold [174]. Santander et al. [175] showed that arbuscular mycorrhizae enhanced the activities of SOD, APX, and CAT in salinity-stressed cucumber (40 and 80 mM NaCl). In *Phaseolus vulgaris* and *Triticum aestivum, Glycyrrhiza glabra* root extract or 6% *Moringa oleifera* leaf extract ameliorated the salinity stress by enhancing the antioxidant activity [176,177]. Lastly, supplementation with penconazole (15 mg/L) modified the CAT, SOD, PPO, and POD expression and antioxidant activity to mitigate the effect of salt stress on *Sesamum indicum* [178]. These results demonstrate the important role played by the antioxidant defense machinery in the mitigation of salt stress.

**Figure 4:**
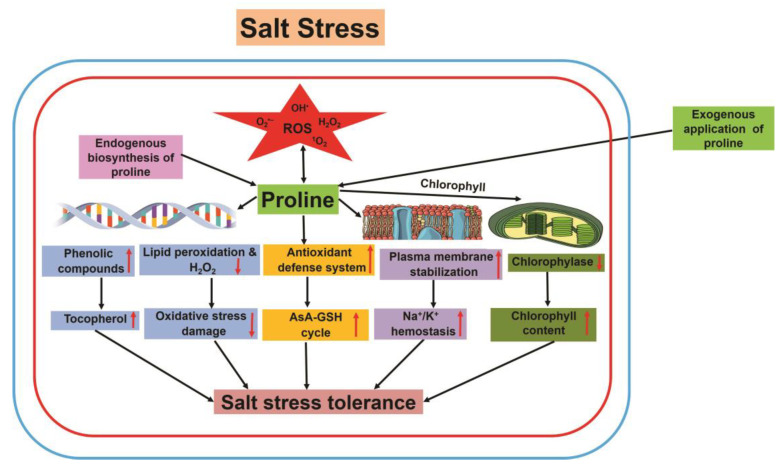
The mechanisms by which exogenous and endogenous proline ameliorate salt stress tolerance in plants. ROS, reactive oxygen species; HO•, hydroxyl radical; H_2_O_2_, hydrogen peroxide; O_2_^•−^, superoxide radical; ^1^O_2_, singlet oxygen; AsA-GSH, ascorbate-glutathione cycle.

Exogenous application of proline enhanced stress tolerance (Figure 4) and plant growth by protecting the structure and function of PSII, reducing lipid peroxidation, and quenching ROS during stress conditions [179]. Proline plays a vital role in plants under different stress conditions, defending the PM from oxidative destruction by enhancing the activities of antioxidant enzymes (including POD and SOD). The increased activities of POD and SOD seem to improve salt stress tolerance in soybeans [180]. Under high salinity stress, the salt-tolerant cultivars of *Cucumis melo* and *Solanum tuberosum* have a higher level of proline than salt-sensitive cultivars [181]. Similarly, salinity-stressed *Solanum lycopersicum* exhibited a higher accumulation of proline than non-stressed *Solanum lycopersicum* [182]. Additionally, external application of proline determined enhanced plant growth in salinity-stressed *Calendula* sp. and *Vicia faba* [183,184]. Conversely, a few studies reported that proline acts as a stress indicator; however, it does not improve salt stress tolerance [185,186]. Usually, proline plays an essential role in preventing oxidative injury of subcellular structures, PM, and proteins by scavenging ROS [187].

The biosynthesis of proline through the glutamate pathway uses two molecules of NADPH for one molecule of proline, removing the electrons from the chloroplast and buffering the redox potential of the cell [188]. Proline accumulation in the leaves under high salinity stress allows the continuous reduction of carbon dioxide by regulating ROS production and preventing photoinhibition. Furthermore, mitochondrial proline is metabolized, and the decreasing power could be dissipated by respiratory electron transport coupled to alternative oxidase to avoid complex III and IV. Studies of the transcriptional regulation of specific genes in knockout mutants of *Arabidopsis* showed that *P5CS1* regulates proline synthesis, with its expression being highly elevated under high salinity stress [189]. Knockout mutations of *P5CS1* lead to a severe decrease in salinity stress-induced proline expression. Subsequently, growth is impaired, and the levels of ROS increase, indicating that these mutants are hypersensitive to high salinity stress [189]. Glycine betaine is an organic osmolyte similar to proline, synthesized by numerous plants to maintain the osmotic equilibrium of the intracellular compartments during high salinity stress [190]. The effect of glycine betaine and proline on the antioxidant defense system during high salinity stress was studied using tobacco cells suspended in bright yellow-2 [191,192]. High salinity stress significantly decreased GSH levels and reduced ABA levels and the activities of water cycle enzymes. The external supplementation of glycine betaine or proline restored the activities of these enzymes [191]. These results demonstrate that proline and glycine betaine regulate the expression and function of the antioxidant enzymes during salt stress.

## 7. Crosstalk between Different Signaling Molecules during Salt Stress

**Figure 5:**
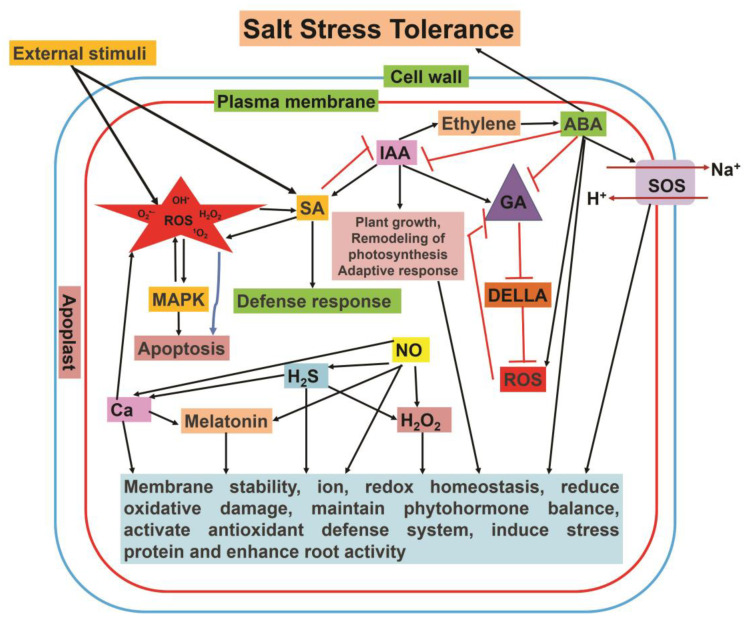
Overview of crosstalk between the different signaling molecules and phytohormones during salt stress tolerance. ROS, reactive oxygen species; O_2_^•−^, superoxide anion; ^1^O_2_, singlet oxygen; HO•, hydroxyl radical; H_2_O_2_, hydrogen peroxide; NO, nitric oxide; H_2_S, hydrogen sulfide; Ca, calcium; SA, salicylic acid; GA, gibberellins; IAA, indole acetic acid; ABA, abscisic acid; SOS, salt overly sensitive; MAPK, mitogen-activated kinase protein.

Salinity tolerance is regulated by an intricate gene network and affects diverse cellular processes in plants. Different signaling molecules such as NO, H_2_O_2_, and other ROS, H_2_S, calcium (Ca^2+^), and phytohormones crosstalk and control diverse biological processes and the expression of genes implicated in salt stress tolerance [9,10,11]. The crosstalk between the different signaling molecules leading to salt stress tolerance is summarized in Figure 5.

### 7.1. H_2_O_2_

H_2_O_2_ formation results in oxidative stress, and its accumulation causes cellular and molecular damage, activating programmed cell death [193]. Its generation is induced by exposure to various types of environmental stress in plants. Additionally, in plants, H_2_O_2_ serves as a signal to regulate various biological processes and the crosstalk among different signaling pathways [194]. H_2_O_2_ and NO signaling pathways are strongly correlated with the plant response to various environmental stimuli [195]. High salinity stress induces alterations in the generation of H_2_O_2_ and NO. Zhao et al. [196] found that high salinity stress decreased the transcript-level expression of *AtNOA1* in Arabidopsis, resulting in a subsequent reduction in NO levels. Conversely, high salinity stress moderately elevated the transcript-level expression of *OsNOA1*, a homolog of AtNOA1 in rice [197]. Furthermore, treating wheat seeds with H_2_O_2_ enhanced salt tolerance [198]. Besides influencing transcription, the crosstalk between NO and H_2_O_2_ also regulates translation and post-translational changes. In Bermuda grass, NO and H_2_O_2_ stimulated the activities of CAT and SOD via ABA [199], affecting the expression and antioxidant activity during high salinity stress [200]. Subsequently, SA induced H_2_O_2_ by regulating the expression of SOD. Thus, SA and H_2_O_2_ work together in a self-amplifying manner. H_2_O_2_ at higher concentrations causes nitrosative or oxidative stress, whereas at basal levels it functions as a signaling molecule, regulating plant response under diverse stress conditions.

### 7.2. NO

NO is a key gasotransmitter that regulates various signaling pathways and a range of physiological processes during salt stress in plants [201]. The crosstalk between NO and different signaling molecules and plant hormones improves salt stress tolerance [202,203]. ABA, auxin, and ET are important phytohormones that travel from the salt-exposed roots to the leaves, enhancing the biosynthesis of NO [204]. Additionally, NO mitigates oxidative damage during salt stress by increasing antioxidant activity and reducing the activity of thiobarbituric acid [205]. In cotton, exogenous application of NO suppressed salt-induced leaf senescence by downregulating the genes associated with ABA biosynthesis (2- and 9-cis-epoxycarotenoid- dioxygenase) [206]. *Arabidopsis* callus exposed to salt stress (100 mM NaCl) triggered the accumulation of NO and a subsequent increase in the expression of H^+^-ATPase in the PM [207]. However, in a suspension culture of salt-stressed tomato (100 and 200 mM NaCl), an antagonistic relationship was observed between the formation of ET and NO. Thus, enhanced synthesis of ET induces the generation of ROS, thereby increasing the ratio of dead cells, while NO production decreases the ratio of dead cells. The cultures of suspension cells and apical root fragment were both deficient in ET and NO-generated ionic imbalance (Na^+^/K^+^), resulting in enhanced susceptibility to salt stress [203]. The supplementation of sodium nitroprusside (SNP), SA, and their accumulation decrease NaCl-stimulated toxicity by increasing proline levels and GPX, CAT, and APX activity in soybean seedlings [208]. SA interacts with the NO signaling cascade, reducing H_2_O_2_ accumulation, which increases the H^+^-ATPase influx into the PM. The combined effect of NO and SA enhances the absorption of Mg^2+^/Ca^2+^ and reduces the uptake of Na^+^ in conditions of salt stress [209].

Sulfur is a primary biocomponent and is present in methionine, GSH, cysteine (Cys), coenzyme A, thioredoxin, sulfo-lipids, and iron–sulfur (Fe–S), which control cellular processes in high salinity conditions [210,211]. Additionally, NO induces S-assimilation, which is associated with ET biosynthesis via the production of cysteine. Under salt stress, NO and sulfur interact to control ET and ABA concentrations in plant cells, ensuring their protection and regulation of stomatal activities and photosynthesis [212]. NO functions as a central regulatory signal, triggering different biochemical activities. Furthermore, its interaction with sulfur assimilation improves salt stress tolerance [212]. NO and other signaling molecules such as H_2_S help counteract the effects of salt stress in plants. In cucumber, NO application under saline stress modulates proline metabolism, increasing the accumulation of free proline ratio; thus, it maintains the turgor potential and protects cucumber seedlings [213]. The exogenous application of sodium nitroprusside and CaCl_2_ improves the salt tolerance of mustard by enhancing the antioxidant activity and the accumulation of proline and glycine betaine. Subsequently, a decline in thiobarbituric acid-reactive substances, electrolyte leakage, and H_2_O_2_ is observed [214]. In *Lactuca sativa*, NaCl application activates ionic, osmotic, and oxidative stress, leading to hormonal imbalances and reduced plant growth. Supplementation of NO results in decreased Na^+^ accumulation and the maintenance of mineral balance, thereby improving the photosynthetic rate and growth [215]. NO signaling interacts with phytohormones, regulating osmotic stress, activating the antioxidant defense machinery, and enhancing salt stress tolerance. *Crocus sativus* treated with NO displays vigorous growth during high salinity. NO application induced the biosynthesis of secondary metabolites, enhanced the deposition of compatible solutes, and enhanced the activities of antioxidant enzymes, while exogenous application of SA did not ameliorate the growth of plants under salt stress [216]. In salt-stressed *Brassica napus* L. seedling roots, concomitant application of NO-releasing substances and of melatonin neutralized the inhibitory effect of NaCl on seedling growth and reestablished redox and ion homeostasis, which was demonstrated by a decrease in the overproduction of ROS, the ratio of Na^+^/K^+^, and the synthesis of thiobarbituric acid-reactive substances. Melatonin inhibited the effects of salinity stress via a NO-dependent mechanism [202]. Thus, NO serves as a positive regulator in a complex signaling network regulating the plant defense machinery during salinity stress.

### 7.3. ROS

Salt overly sensitive (SOS) pathway plays a crucial role in the response to salt stress. It regulates the membrane conductance in the epidermal cells of the root when exposed to high salinity. Thus, it helps detoxification by the epidermal cells of the root by removing the ions from the root [217]. The SOS pathway is key to maintaining ion homeostasis when plants are exposed to salt stress [218]. During salt stress, the generation and quenching of ROS act as distress indicators. Maintaining cellular redox homeostasis and adequate concentrations of antioxidants is critical for ensuring adequate levels of ROS, which participate in stress perception, activate downstream signaling pathways, and determine plant adaptation and response to salt stress. Nevertheless, accumulation of ROS in plants impairs essential metabolic pathways [14,31]. Oxidants release electrons, which act as a signaling clue, alerting plant cells that they need to adapt to the high salinity conditions [19]. Salt stress also causes ROS-mediated harm to nucleic acids, proteins, and lipids, leading to apoptosis [203]. However, Ca^2+^ and ROS are well-known intracellular signals. Under severe salt stress conditions, the concentration of cytosolic Ca^2+^ is significantly elevated, activating specific signaling pathways [36]. Salt stress tolerance in plants is also ensured by mechanisms such as regulation of ion transport including K^+^ and Na^+^, accumulation of compatible solutes, and an increase in the expression of stress-related genes [219].

The production of ROS in plant organelles is unavoidable as they are byproducts of metabolic processes [17,19,31]. However, oxidative stress-induced programmed cell death via apoptosis is avoidable and preventable by antioxidant enzymes. Numerous plants often go through necroptosis, even in the absence of a stressful environment [220]. Singlet oxygen is used as a substrate by LOX to prompt a metabolic cascade that generates essential stress signals such as JA. Additionally, ROS stimulate ABA biosynthesis [5]. Salt stress determines the closure of the stomata, decreasing moisture loss and the influx of CO_2_. Consequently, reduction of carbon and utilization of photosynthetic NADPH in the Calvin cycle decrease, leading to electron holes in PS I and electrolyte leakage [165]. Glycolate oxidase is the main source of ROS in peroxisomes in both normal and stress conditions [51]. The quenching of ROS can be achieved by various components, including NO, which abolishes oxidative stress by activating SOD and increasing the concentrations of GSH; thus, it interferes with the effect of oxidizing factors in restoring redox homeostasis [212]. Other signaling molecules contributing to the regulation of oxidative stress in various plant species include H_2_S and GSH, which crosstalk with NO [196,212]. Phytohormones influence the production of NO, which acts as an intermediate between different signaling pathways. ROS formed by NADPH oxidase in the PM activate the ABA signaling pathway in plants [221]. The negative effect of ROS on stress-induced protein aggregation is influenced by the capacity of ROS recycling via the scavenging system. The accumulation of diverse forms of ROS contributes to the adaptation to stress conditions and programmed cell death. The exogenous application of SA via the root resulted in an increased concentration of H_2_O_2_ in the root tissue and young leaves, which killed the plant [208]. Szepesi et al. [222] reported that in salt stress conditions, plants supplemented with SA contain more Na^+^ than non-supplemented plants. Furthermore, leaves did not display any symptoms of salt injury, and the integrity of the membrane was maintained. Owing to the above-mentioned roles of abiotic-stress-induced intracellular ROS, a question was raised on whether intracellular amounts of NO and ROS can contribute to the protection of the distressed leaf protoplasts. ROS also activate Ca^2+^-permeable channels such as respiratory burst oxidase homolog (Rboh), which is assumed to represent a positive feedback loop that induces Ca^2+^ and ROS signals in root cells. In *Arabidopsis,* RHD2/RbohC influenced the generation of ROS, regulating root hair elongation in a Ca^2+^-dependent manner [223].

Salt stress increases cytosolic Ca^2+^ levels via depolarization and activation of hyperpolarization-activated Ca^2+^ permeable channels in the PM. This increase plays an important role in ROS signaling and salt stress tolerance [36]. Besides increasing ROS levels, salt stress stimulates the synthesis of polyamines. The polyamines and hydroxyl radical (HO•) might eventually influence the Ca^2+^ pathway in plants [224]. This could be a unique mechanism by which ROS increase salt stress acclimation. Xu et al. [225] demonstrated that the Ca^2+^/Calmodulin-dependent Protein Kinases (CDPKs) were more evident in halophytes compared with glycophytes under salt stress. The calcium-binding proteins play an important role as amplifiers in early calcium influx during salinity stress. Ca^2+^ signaling has a significant impact on signaling mechanisms in salt stress. Two-pore channel 1 is involved in the dissemination of the signal (Ca^2+^) in salt stress and the activation of the plant defense [226]. Furthermore, NO manifests potential antioxidant activity by minimizing and inhibiting protein oxidation and lipid peroxidation. In salt stress, an elevated level of NO minimizes salt stress-induced injuries [212]. The protective role of NO during salt stress is ensured by the enhancement of the antioxidant activity in different plant species [129,212,215].

### 7.4. H_2_S

H_2_S serves as a signaling molecule and plays an important role in seed germination, postharvest senescence, and adventitious rooting. Furthermore, it also has a protective role in multifactorial stress conditions, including biotic and abiotic stresses [74,174]. It increases salt tolerance by increasing the content of chlorophyll and soluble proteins and preventing the accumulation of ROS [227]. Furthermore, H_2_S donors such as phosphinodithioate, morpholin-4-ium 4 methoxyphenyl, NOSH-aspirin, CaS2, AP39, sodium hydrosulfide, diallyl trisulfide, and dialkyldithiophosphate, have been identified and characterized. NOSH-aspirin simultaneously released two gasotransmitters, for instance, NO and H_2_S [228]. Furthermore, the levels of ROS are controlled by two mechanisms: scavenging the surplus of ROS by non-enzymatic antioxidants (ascorbate and glutathione) and by antioxidant enzymes associated with the AsA-GSH cycle. In Chinese cabbage, the accumulation of ROS was minimized by the exogenous application of H_2_S, as this enhanced the activity of the antioxidant enzymes CAT and SOD [229]. Hence, we can speculate that the activity of antioxidant enzymes is regulated by H_2_S through post-translational changes. By regulating the antioxidant defense system, H_2_S also preserves membrane integrity and ROS homeostasis, increasing the tolerance level under saline conditions. Mostofa et al. [227] demonstrated that H_2_S contributes to the maintenance of the Na^+^/K^+^ ratio during salt stress. In rice, salt stress increases Na^+^ concentration and decreases K^+^ concentration, resulting in an increased Na^+^/K+ ratio in the root and leaves. Exogenous application of H_2_S maintained K^+^/Na^+^ homeostasis during salt stress. Furthermore, under high salinity stress, H_2_S acts synergistically with NO in response to salt stress [230]. The downstream and upstream connection between NO and H_2_S revealed that in stressful conditions, NO induces H_2_S accumulation in plants and increases stress tolerance; H_2_S further functions as a downstream signal. Nitrosothiol is a newly identified signal molecule generated when H_2_S responds to NO Cys and plant hormones and contributes to NO-mediated salt stress tolerance.

### 7.5. Ca^2+^

Calcium is one of the key signaling molecules and secondary messengers that crosstalks with other signaling molecules to minimize the impact of salt stress. The activities of phenylalanine ammonia lyase (PAL) and flavonoids were increased after the incorporation of calcium chloride or an ionophore into the culture medium [231]. Further, Ca^2+^ also mediates the production of special metabolites induced in response to SA, ABA, and JA [36,170,222]. The functions of Ca^2+^ and MT—and how they influence the development of phenolic compounds under salt stress—were examined in *Dracaena kotschyi*. Exogenous application of NaCl decreases the dry biomass of shoots; however, it enhances H_2_O_2_ production, electrolyte leakage, the scavenging capacity of 2,2-diphenyl-1-picrylhydrazyl, and the expression of *TAL*, *PAL*, and *RAS* (compared with non-exposure to high salinity). Pretreatment of *D. kotschyi* with an inhibitor of the melatonin pathway had no influence on the Ca^2+^-induced generation of phenolic compounds in salt-treated plants [232]. However, treatment of *D. kotschyi* with a PM channel blocker during salt stress had the opposite effect. These results indicate that melatonin induces the biosynthesis of phenolic compounds only when there is an influx of Ca^2+^.

NO and H_2_O_2_ work together with Ca^2+^ to form an intricate regulatory network to mitigate the effect of salinity stress [194,230,233]. Hajihashemi et al. [233] reported that pretreatment of quinoa seeds with calcium chloride, H_2_O_2_, and sodium nitroprusside exhibits a positive linear relationship with the germination rate and germination index, while under salt stress, a negative linear relationship was observed with the mean germination time. Pretreatment of seeds enhanced their germination and the quick establishment of the seedling in the saline soil. Additionally, pretreatment with NaCl reduced α- and β-amylase activities. This inhibited starch hydrolysis, impairing seed germination. The above-mentioned pretreatment reduced the negative effect on these enzymes. Another study demonstrated that external application of calcium chloride and H_2_O_2_ might decrease the harmful effect of multifactorial abiotic stress on the activity of amylase and rescue seed germination [234]. Interestingly, Hajihashemi et al. [233] observed that the existence of Ca^2+^, NO, and H_2_O_2_ induced the activity of amylase. The increased starch degradation enhanced seed germination and sprouting in quinoa, minimizing the negative impact of salt stress.

Polyamines such as putrescine, spermine, and spermidine have been implicated in a wide range of plant developmental processes. They also regulate the adaptive response to various stress conditions [235]. Additionally, polyamines play an essential role in apoptosis. Stress conditions significantly alter the expression of genes involved in polyamine biosynthesis [236]. The differential response of the arginine decarboxylase gene in the biosynthesis of polyamines contributes to the acclimation of plants during salt stress. The analysis of genes involved in the biosynthesis of polyamines in different rice cultivars showed that transcript-level expression of *ADC* was elevated under salt stress conditions (compared with normal conditions) [237]. The metabolic pathway of polyamines crosstalks with various signaling pathways such as those of gamma-aminobutyrate (GABA), H_2_O_2,_ and ABA [171,235,237]. GABA improved the salt tolerance in *Lactuca sativa* [238]. Additionally, polyamines also increase the production of NO and its crosstalk with various stress intermediaries, including protein kinases and Ca^2+^. They regulate ion channel activity by modulating their conductivity under salt-stress conditions [239]. Moreover, polyamines control the activities of different ion channels indirectly by increasing the binding of 14–3–3 proteins. Elevated levels of cytoplasmic Ca^2+^ might have a detrimental effect on the standard cellular metabolism in stressful conditions. Polyamines trigger the efflux of Ca^2+^ by activating the membrane Ca^2+^-ATPase and the PM channel. Thus, they play an important role in maintaining intracellular Ca^2+^ homeostasis and sustaining a steady plasmatic level [224]. However, the underlying mechanism linking polyamine metabolism and Ca^2+^ signaling is poorly understood.

Numerous studies have demonstrated the tight regulation of NO metabolism under salt stress [212,216]. In plants and animals, NO is mainly produced by NO synthase [235]. Its production is correlated with polyamines through the common precursor L-arginine. Thus, polyamines, including spermine and spermidine, might be involved in the generation of NO in plants [240]. Additionally, the function of NO in signaling might be affected by the movement of intracellular Ca^2+^. Furthermore, it may influence calcium ion channels, eventually activating Ca^2+^ signaling. The mechanisms underlying this crosstalk are largely unknown. The activation of NO synthase, which catalyzes NO synthesis, involves activation of calmodulin and Ca^2+^ signaling [197,206]. Moreover, NO plays a critical role in the activation of PM channels, following the release of Ca^2+^ during salt stress recovery [204]. Therefore, Ca^2+^ signaling plays an essential role in the response and adaptation of plants under salt stress.

### 7.6. Phytohormones

In agriculture worldwide, the quality and yield of crops are significantly affected by high salinity stress. The capacity of plants to tolerate this type of stress can be enhanced by the exogenous application of H_2_O_2_, NO, and H_2_S. For instance, supplementation of NO in salinity-stressed lettuce reduces the accumulation of Na^+^ and regulates the concentrations of mineral nutrients, thereby maintaining the photosynthetic rate and restoring vegetative growth. Furthermore, NO also restored the osmotic balance, stimulated the antioxidant system, and reinstalled the equilibrium of plant hormones, improving the plant’s tolerance to high salinity stress [215]. High salinity stress impairs plant development by increasing the levels of ABA and MDA and the expression of proline-encoding genes and reducing electrolyte leakage and the K^+^/Na^+^ ratio. Exogenous application of 24-epibrassinolide, sodium nitroprusside (SNP), or their combination enhanced endogenous ABA levels in *Brassica juncea* via nitrogen and proline metabolism [241]. High salinity stress has a detrimental effect on plants via a shortage of water and a decrease in the K^+^/Na^+^ ratio. By generating ROS such as hydroxyl radicals (HO•), superoxide (O_2_^−^•), and H_2_O_2_, it also modulates the cellular redox pathways. The formed free radicals negatively impact cellular processes by inducing lipid peroxidation and damaging nucleic acids and proteins. Therefore, they impair fundamental processes such as plant growth and development, gaseous exchange, nitrogen, carbon, and proline metabolism [241,242]. The crosstalk under high salinity stress between phytohormones and other signaling molecules is presented in Table 2. NO controls the homeostasis of the ABA and different biochemical pathways—including leaf senescence, seed germination, maturation, dormancy, seedling growth, regulation of stomata, fruit ripening, and response to diverse biotic and abiotic types of stress—in plants. Furthermore, NO is implicated in post-translational modifications, including nitration and nitrosylation of sulfur and tyrosine residues of proteins that control ABA signaling in plants. NO impaired the plant antioxidant machinery by altering the levels of ascorbate, GSH, CAT, and SOD and also influenced the ABA-mediated ROS production [243]. Phytohormones play a crucial role in adjusting to harsh environment conditions including salt stress by altering the physiological response of plants [3,5,244]. Gibberellins (GAs) and ET play a critical role in alleviating salt stress by inducing the expression of stress-related genes and enhancing plant growth. GAs and ET are interrelated, as GA induces the biosynthesis of ET and GA signaling relies on ET [245]. Furthermore, whole-genome transcriptome profiling studies revealed that in plants, salt stress significantly upregulated genes related to GA and ET metabolism. The precursor of ET, 1-aminocyclopropane-1-carboxylic acid (ACC), might also be involved in the biosynthesis of GA. Exogenous application of GA3 and ethephon suppressed the negative effect of salt stress on seed germination in *Amaranthus caudatus* [246]. Furthermore, ethephon exhibited a more stimulatory effect on seed germination than GA3 during salt stress. In pea, ET synthesis was negatively regulated by other plant hormones. The study also revealed that ET inhibits the production of GA [247]. In *Arabidopsis*, GA and ET positively regulate hypocotyl elongation. GA alone is inefficient; however, it acts synergistically with ET and enhances the growth of emerging roots. These results suggest that GA and ET do not have additive results but rather act synergistically.

In plants under salt stress, seed germination is influenced by NO and RT. In *Arabidopsis*, the exogenous application of sodium nitroprusside and ACC counteracts the negative impact of salt stress on seed germination [248]. Conversely, their stimulatory effect is repressed by NO or 2-phenyl-4,4,5,5-tetramethyl-imidazoline-1-oxyl-3-oxide. These results suggest that ET and NO have a combined effect on seed germination during salt stress. Additionally, the production of NO was induced by ACC. *ACS2* expression was elevated by sodium nitroprusside. These findings demonstrate the significance of NO and ET during salt stress. Supplementation of ACC enhances seed germination under oxidative stress; seed germination is activated by H_2_O_2_. Nevertheless, NO-treated *Arabidopsis* was not substantially affected, while the seeds of the ET-insensitive mutant were not affected in any way.

SA plays a crucial role in various physiological and biochemical processes and has significant effects on the tolerance to biotic and abiotic stress [249,250,251]. It acts as an endogenous signal molecule and activates the defense machinery in plants [252]. It significantly improves seed germination under salinity stress; furthermore, in *Matricaria chamomilla*, external application of SA (500 μM/L) markedly enhanced plant growth under salt stress and normal conditions [253]. However, some studies report contradicting results. For instance, Li et al. [254] and Arfan et al. [255] showed that foliar spray of SA prevented salinity stress-induced growth inhibition at certain concentrations of salt, while no improvement was reported at higher levels of salinity stress. Cao and colleagues [256] reported that insufficient levels of SA can protect against modest salinity stress in *Arabidopsis*. These findings demonstrate that the beneficial effect of SA on salinity stress might depend on the concentration of salt and may vary between different plant species. The detailed mechanism underlying the effect of SA on plants exposed to salt stress remains unknown.

Auxin regulates diverse physiological and developmental processes in plants [257,258,259]. Reduced levels of auxin are linked with decreased distribution and transport along the root [260,261]. In normal conditions, auxin mediates the development of the lateral root, which is impaired in response to higher concentrations of salt [262]. Song et al. [263] reported that almost all *OsIAAs* respond to different abiotic stresses, suggesting a link between abiotic stresses and plant growth. Additionally, the expression of *OsIAA18* in rice was induced by phytohormones and various abiotic stresses, including high salinity, drought, and treatment with abscisic and IAA treatments [263]. Overexpression of *OsIAA18* enhanced the tolerance to salinity and osmotic stress in *Arabidopsis* [264]. Overexpression of *OsIAA6/OsIAA20* improves the tolerance to salinity and drought in rice [265,266], while the overexpression of *VvIAA18* increases the tolerance to salinity in tobacco [267]. Therefore, the effect of high salinity stress (or various types of abiotic stress) on other genes related to auxin transport should be elucidated.

## 8. Transgenic Approach to Improve the Antioxidant System in Plants under Salt Stress

**Figure 6:**
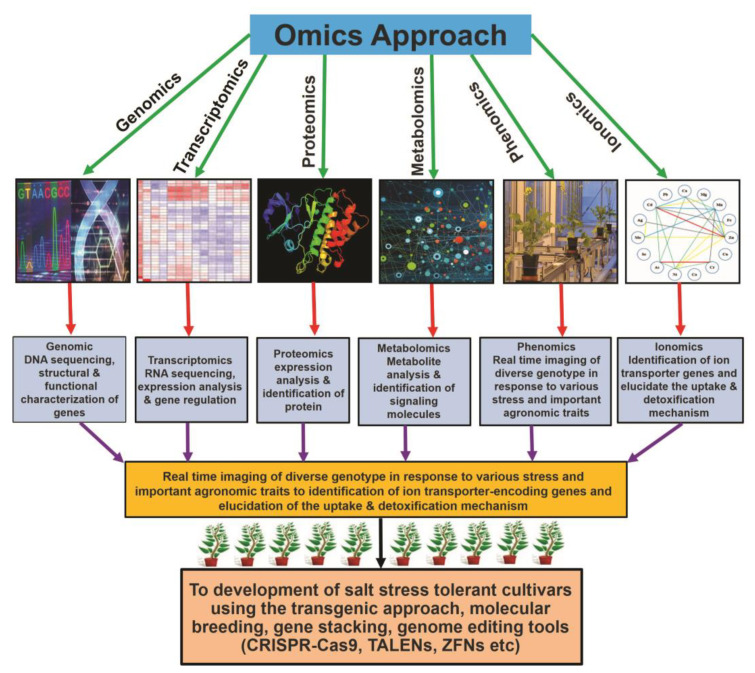
Utilization of different “-omics” approaches to develop salt-tolerant cultivars for improving the growth and productivity of different plant species.

In the past few years, biotechnology approaches have been extensively used to develop cultivars with better nutritional quality, yield, and stress tolerance. Omics are the most prominent and efficient approaches to improving plant tolerance against various biotic and abiotic stresses. Plant biologists can utilize the omic tools, including genomics, transcriptomics, proteomics, metabolomics, phenomics, and ionomics, to identify genes, RNAs, proteins, metabolites, phenotypes, and ions belonging to the intricate regulatory mechanisms activated during the response to internal and external stimuli and during various developmental stages of plants (Figure 6).

Furthermore, deciphering the underlying mechanisms by which plants perceive and transduce signals that initiate adaptive responses is a prerequisite for the identification of key genes and pathways to cope with future challenges. Furthermore, decoding and understanding such a regulatory network of genes would help gain insight into plant biology and could be practically applied to improve the quality, productivity, resistance to disease, and stress resistance of crops. This will allow us to feed the ever-increasing human population. Hence, plants can be genetically engineered to increase their tolerance to oxidative stress and improve the response and activities of the antioxidant defense system. An overview of genetically engineered plants with increased activities of the antioxidant enzymes under salt stress is presented in Table 2. Transgenic *Chrysanthemum* overexpressing *DgNAC1* exhibited improved salt tolerance by reducing the content of O_2_^−^•, H_2_O_2_, and MDA and increasing the activities of CAT, POD, and SOD [268].

**Table 2:** Genetic engineering methods—recent advances to improve the antioxidant defense machinery under salt stress.

Gene Name	Source of Genes	Transgenic Plants	Stress Characteristics (Concentration of Salt Solution and Duration of Exposure)	Mode of Action	Reference
*DgNAC1*	*Dendronthema grandiflorum*	*Chrysanthemum*	100, 200, and 400 mM NaCl; 1, 5, 10, 15 days	Reduced levels of O_2_^−^•, H_2_O_2_, and MDA; increased activities of CAT, POD, and SOD	[268]
*RaAPX* and *PaSOD*	*Rheum australe* and *Potentilla atrosanguinea*	*Solanum tuberosum*	0, 50, 100, and 150 mM NaCl; 7 and 15 days	Enhanced activities of SOD and APX in both the transgenic variants of potatoes	[269]
*OsMYB6*	*Oryza sativa*	*Oryza sativa*	150 mM NaCl; 6 days	Increased concentration of proline, elevated activities of SOD and CAT, decreased content of MDA and REL	[270]
*StCYS1* in	*Solanum tuberosum*	*Solanum tuberosum*	0.17 mol/L NaCl; 0, 3, 5, and 7 days	Enhanced accumulation of proline and increased scavenging of H_2_O_2_	[271]
*OsSTAP1*	*Oryza sativa*	*Oryza sativa*	150 mM NaCl; 5 days	Increased activities of SOD, POD, and CAT	[272]
*DnWRKY11*	*Dendrobium nobile*	*Nicotiana tabacum*	200 mM NaCl; 20 days	Enhanced activities of SOD, CAT, POD; reduced content of MDA	[273]
*GmMYB84*	*Glycine max*	*Glycine max*	150 and 200 mM NaCl; until the seed germination	Higher activities of antioxidant enzymes POD, CAT, and SOD and accumulation of proline	[274]
*OsEXPA7*	*Oryza sativa*	*Oryza sativa*	150 mM NaCl; two to three weeks	Increased activity of SOD and POD, reduced accumulation of ROS and MDA, increased accumulation of proline	[275]
*MsWRKY11*	*Medicago sativa*	*Glycine max*	100 and 200 mM NaCl; 7 days	Increased contents of soluble sugar, chlorophyll, proline; enhanced activities of CAT and SOD; reduced contents of O_2_^−^•, H_2_O_2,_ MDA	[276]
*nbexo70d1*	*Nicotiana benthamiana*	*Nicotiana benthamiana*	100, 200, and 300 mM NaCl; 5 days	Declined accumulation of ROS and decreased activity of NADPH oxidase	[277]
*VvIAA18*	*Vitis vinifera*	*Nicotiana tabacum*	200 mM NaCl; every 2 days for 8 weeks	Induced the expression of salt stress-responsive genes LEA5, P5CS, POD, and SOD; increased activities of POD and SOD	[278]
*VvWRKY30*	*Vitis vinifera*	*Arabidopsis thaliana*	150 mM NaCl; 3, 6, 9, 12, and 24 h	Increased activities of antioxidant enzymes CAT, SOD, and POD	[279]
*SsMAX2*	*Sapium sebiferum*	*Arabidopsis thaliana*	100 and 150 mM NaCl; 15-day-old seedlings	Enhanced activities of SOD, POD, and APX	[280]
*MfWRKY70*	*Myrothamnus flabellifolia*	*Arabidopsis thaliana*	50, 100, or 150 mM NaCl; treated to seed	Reduced levels of H_2_O_2_ and differential activities of POD, SOD, and CAT	[281]
*PeHSF*	*Populus euphratica*	*Nicotiana tabacum*	150 mM NaCl; 1, 8, 15, and 23 days	Increased activities of APX, GPX, and GSH	[282]
*ThHSFA1*	*Tamarix hispida*	*Populus trichocarpa*	200 mM NaCl; every 2 days for 10 days	Reduced levels of ROS, increased activities of antioxidant enzymes	[283]

Similarly, transgenic potatoes overexpressing *PaSOD* (*Potentilla atrosanguinea*) and *RaAPX* (*Rheum austral*) displayed increased activity of APX and SOD. APX and SOD might be functioning as positive regulators, enhancing salt tolerance by the regulation of ROS signaling and by the stimulation of lignin biosynthesis [270]. Rice overexpressing *OsMYB6* exhibited enhanced tolerance to salt and drought stress compared with control rice. Under salt and drought stress, transgenic plants showed higher proline levels, higher SOD and CAT activities, and lower MDA and REL levels than wild-type plants [271]. Potatoes overexpressing *StCYS1* had a significantly higher salt tolerance than wild-type potatoes. Transgenic potatoes presented higher levels of proline, better scavenging of H_2_O_2_, and a superior integrity of the cell membrane than non-transgenic plants [271]. Rice overexpressing *OsSTAP1* presented enhanced salt stress tolerance, demonstrating higher CAT, POD, and SOD activities and lower Na^+^/K^+^ ratios in the shoots than wild-type rice [272]. Xu et al. [273] overexpressed *DnWRKY11* in tobacco; transgenic tobacco displayed longer root length, a higher germination rate, higher CAT, SOD, and POD activities, and lower MDA concentrations than wild-type tobacco. Soybean plants overexpressing *GmMYB84* displayed a higher germination rate, longer root elongation, higher antioxidant enzyme activities, and higher proline content compared with wild-type plants [274].

Overexpression of *OsEXPA7* significantly increased the salt tolerance of rice. Transgenic rice presented reduced levels of Na^+^ and increased levels of K^+^ in the leaves and roots of the plant. Compared with wild-type rice, transgenic rice presented higher activity of antioxidant enzymes (POD and SOD), lower accumulation of ROS and MDA, and higher proline accumulation. These findings indicate that *OsEXPA7* plays a critical role in enhancing the tolerance to salt stress by regulating the transport of Na^+^ and scavenging ROS [275]. Under high salinity stress, transgenic plants overexpressing *MsWRKY11* showed a better tolerance to salt stress and longer hypocotyls than non-transgenic plants. Additionally, transgenic plants had higher contents of soluble sugar, chlorophyll, proline, CAT, and SOD, lower contents of O_2_^−^•, H_2_O_2_, and MDA, and lower relative electrical conductivity [276]. Tobacco overexpressing *NbExo70D1* had lower accumulation of ROS than control. Pretreatment with diphenylene iodonium, an inhibitor of NADPH oxidase, caused a reduction in salt stress-induced ROS production in the roots of both transgenic and wild-type plants. However, the activity of NADPH oxidase was lower in the transgenic plants than in wild-type plants under salt stress, suggesting *NbExo70D1* participates in NADPH oxidase-mediated production of ROS [277]. Further, Li et al. [278] overexpressed *VvIAA18* in tobacco and *E. coli*; salt stress tolerance significantly improved in transgenic tobacco and *E. coli*. Quantitative reverse transcription PCR revealed that overexpression of VvIAA18 induced the expression of salt stress-responsive genes, such as LEA5, P5CS, POD, and SOD, under salt stress. In addition, transgenic tobacco had higher activities of POD and SOD and higher levels of proline and lower levels of MDA and H_2_O_2_ than wild-type tobacco. Zhu et al. [279] overexpressed *VvWRKY30* in *Arabidopsis.* This significantly improved the salt stress tolerance at different developmental stages, increased antioxidant activity, and decreased the ROS levels. Additionally, the concentrations of soluble sugar and proline and the transcript-level expression of genes involved in antioxidant biosynthesis and proline synthesis were also higher in salt-stressed transgenic plants than in wild-type plants. Overexpression of *SsMAX2* in *Arabidopsis* resulted in decreased degradation of chlorophyll and enhanced accumulation of proline and soluble sugar. The antioxidant activities of SOD, APX, and POD were higher in transgenic plants than in wild-type plants, resulting in a significant decrease in H_2_O_2_ levels [279]. Xiang et al. [280] overexpressed *MfWRKY70* in *Arabidopsis,* significantly improving the tolerance to salt, osmotic, and drought stress, enhancing the root growth, increasing the antioxidant activities of CAT, SOD, and POD, and maintaining ROS homeostasis. Tobacco overexpressing *PeHSF* displayed better control of the ROS homeostasis under salt stress than wild-type tobacco. Transgenic plants showed increased activities of APX, GPX, and GSH. These results indicate that *PeHSF* plays an essential role in the detoxification of ROS and in the transactivation of antioxidant genes under high salinity stress [281]. Transgenic poplar plants overexpressing *ThHSFA1* exhibited increased tolerance to salt stress, reduced ROS levels, and enhanced antioxidant enzyme activities under salt stress [282]. Moreover, compared with wild-type tomatoes, tomatoes overexpressing *SlGGP-LIKE* presented higher levels of reduced AsA, the xanthophyll cycle, and the AsA/DHA ratio in normal conditions, and higher levels of reduced AsA, reduced GSH, total GSH, higher AsA/DHA and GSH/GSSG ratios, and the xanthophyll cycle under salt stress conditions. Thus, transgenic plants showed better salt stress tolerance, higher photosynthetic, antioxidant, and D1 protein activities, and lower levels of ROS and membrane damage than wild-type plants. Overexpression of *SlGGP-LIKE* increases tolerance to salt stress by inducing the synthesis of AsA in tomatoes [283]. Taken together, these results demonstrated that transcriptional factors might be prominent candidates to develop genetically engineered cultivars with increased salt stress tolerance. However, rigorous screening and time-consuming preliminary studies are required before leveraging these genes for generating salt-stress-tolerant cultivars.

## 9. Conclusions and Future Perspectives

The frequency and intensity of multifactorial stresses have perturbed plant health through ROS generation. Changing climate is a major challenge for food security, particularly when considering the ever-growing world population. Furthermore, ROS are a double-edged sword. Basal concentrations activate signaling pathways that sustain cellular homeostasis and help plants adapt to diverse stresses; however, when the concentration of ROS surpasses a particular threshold, oxidative stress is triggered. Antioxidants are essential to maintain the equilibrium between the generation of ROS and their quenching, reducing the impact of stress. Inventing techniques that prevent the ROS-induced losses during stress and elucidating the underlying mechanism will contribute to the development of crops adapted to environmental conditions. Recently, molecular priming has shown huge potential to enhance plant tolerance against environmental stresses; however, some gaps in knowledge still exist. Furthermore, identification of putative candidate genes, proteins, and metabolites regulating diverse signaling pathways using the “-omics” approach will help find new approaches (Figure 6). Remarkably, the identification and mapping of quantitative trait loci will contribute to our understanding of the intricate regulatory network of genes and metabolites. Additionally, the impact of crosstalk between different signaling molecules and phytohormones is critical for developing plants tolerant to diverse types of stresses. Thus, there is an urgent need for intensive research on the generation of climate-smart crops through the application of different molecular biology techniques, including CRISPR/CAS. Moreover, elucidating and improving the functioning of the plant regulatory networks and their effective integration with crop modeling and phenomics are crucial to achieving the sustainability of ecosystems in changing climate scenarios. Integrated research projects can contribute to the generation of climate-smart crops with lifelong tolerance to stress, thereby increasing agricultural production and productivity.

## Figures and Tables

**Table 1:** Different types of ROS (^1^O_2_, HO•, O_2_^−^•, and H_2_O_2_), their place of production, mechanisms of action, and scavenging systems.

Types of ROS	Sites of Production	Mechanisms of Action	Radical Type	Scavenging System
Hydroxyl radical (HO•)	Mitochondria, chloroplasts, and plasma membranes	Reacts with all biomolecules: proteins, lipids, RNA, and DNA.	Free radical	Sugars, flavonoids, proline, and ascorbate
Singlet oxygen (^1^O_2_)	Nucleus, chloroplasts, mitochondria, and plasma membranes	Oxidizes proteins containing cysteine, methionine, tryptophan, tyrosine, and histidine residues; polyunsaturated fatty acids such as methylene-interrupted polyenes and others; and guanine residues of DNA.	Non-radical	α-tocopherol and carotenoids
Superoxide (O_2_^•−^)	Mitochondria, peroxisomes, chloroplasts, electron transfer chains, and apoplast	Dismutates to H_2_O_2_ and reacts with double-bond-containing proteins such as iron-sulfur proteins via the iron atom.	Free radical	Flavonoids, ascorbate, and superoxide dismutase
Hydrogen peroxide (H_2_O_2_)	Mitochondria, peroxisomes, chloroplasts, cytosol and apoplast	Reacts with DNA. Oxidizes proteins and forms HO• and O_2_^−^•. It further reacts with proteins by attacking methionine and cysteine residues. Reacts with heme proteins.	Non-radical	Catalase, ascorbate peroxidase, guaiacol peroxidase, peroxiredoxins, glutathione, and ascorbate

## Data Availability

Data is available in the manuscript.
